# The Prophylactic and Multimodal Activity of Two Isatin Thiosemicarbazones against Alzheimer’s Disease In Vitro

**DOI:** 10.3390/brainsci12060806

**Published:** 2022-06-19

**Authors:** Barbara Mavroidi, Archontia Kaminari, Dimitris Matiadis, Dimitra Hadjipavlou-Litina, Maria Pelecanou, Athina Tzinia, Marina Sagnou

**Affiliations:** 1 Institute of Biosciences & Applications, NCSR “Demokritos”, Patr. Grigoriou and Neapoleos 27, 15310 Athens, Greece; bmavroidi@bio.demokritos.gr (B.M.); matiadis@bio.demokritos.gr (D.M.); pelmar@bio.demokritos.gr (M.P.); atzin@bio.demokritos.gr (A.T.); 2 Department of Pharmaceutical Chemistry, School of Pharmacy, Aristotle University of Thessaloniki, 54124 Thessaloniki, Greece; hadjipav@pharm.auth.gr

**Keywords:** isatin thiosemicarbazones, Akt, GSK-3β, Aβ cytotoxicity, acetylcholinesterase inhibitors

## Abstract

Alzheimer’s disease (AD) is a multifactorial disorder strongly involving the formation of amyloid-β (Aβ) oligomers, which subsequently aggregate into the disease characteristic insoluble amyloid plaques, in addition to oxidative stress, inflammation and increased acetylcholinesterase activity. Moreover, Aβ oligomers interfere with the expression and activity of Glycogen synthase kinase-3 (GSK3) and Protein kinase B (PKB), also known as AKT. In the present study, the potential multimodal effect of two synthetic isatin thiosemicarbazones (ITSCs), which have been previously shown to prevent Aβ aggregation was evaluated. Both compounds resulted in fully reversing the Aβ-mediated toxicity in SK-NS-H cells treated with exogenous Aβ peptides at various pre-incubation time points and at 1 μM. Cell survival was not recovered when compounds were applied after Aβ cell treatment. The ITSCs were non-toxic against wild type and 5xFAD primary hippocampal cells. They reversed the inhibition of Akt and GSK-3β phosphorylation in 5xFAD cells. Finally, they exhibited good antioxidant potential and moderate lipoxygenase and acetylcholinesterase inhibition activity. Overall, these results suggest that isatin thiosemicarbazone is a suitable scaffold for the development of multimodal anti-AD agents.

## 1. Introduction

Alzheimer’s disease (AD) is a neurodegenerative disease associated with neuronal malfunction and death resulting from the β-amyloid peptide (Aβ) misfolding and self-assembling into aggregates outside the neurons. These aggregates primarily disrupt the cell-to-cell communication process and contribute to the blockage of nutrient transport into the neurons [1]. They are the result of a nucleation dependent polymerization process starting from Aβ monomers to oligomers, protofibrils and finally fibrils [2]. Accumulated evidence suggests that soluble Aβ oligomers are the most neurotoxic form of Aβ [3,4], displaying a broad spectrum of pathogenic impacts [5,6].

Aβ deposition, synaptic dysfunction, and neuronal loss in AD has also been closely associated with Glycogen synthase kinase 3β (GSK-3β) overexpression and/or activation [7]. More specifically, the activity of GSK-3β was found to be enhanced upon Aβ treatment via the phosphatidylinositol 3-kinase (PI3K)/Protein kinase B (AKT) phosphorylation of the serine 9 residues signaling pathway, while the suppression of its activity, or its expression, prevented Aβ-mediated neurotoxicity [8]. Moreover, GSK-3β overexpression has been shown to promote neuronal apoptosis in vitro and in vivo [9], GSK-3β inhibition robustly decreased the oligomeric Aβ load in the mouse brain and GSK-3β activation mediates β-amyloid induced neuronal damage [10].

AD is a multifactorial disorder in which Reactive Oxygen Species (ROS) and inflammation are strongly implicated. Chronic inflammation is a common background phenomenon in neurodegenerative diseases, including AD. More specifically, it has been shown that the key regulatory enzymes in the eicosanoid pathway i.e., COX-2, 5-LOX and 12-, and 15-LOX, appear to have an important role in mediating pro-inflammatory responses in AD [11]. Oxidative stress and inflammatory reactions have been involved in the up-regulation of 12/15-LOX activity and expression levels. Regulation of these pathways could therefore be considered for therapeutic purposes [11,12].

Additionally, free radicals are associated in a causative manner with the elevated levels of protein oxidation, lipid peroxidation products and oxidative damage to mitochondria in the AD brain. Hence, antioxidants have been suggested to prevent and offer therapeutic effects on inflammation-generated oxidative stress caused by the lesions of AD [13].

Finally, another molecular characteristic of AD is the decreased level of the neurotransmitter acetylcholine (ACh), vital in maintaining normal cognitive function (cholinergic hypothesis) [14]. As a result, inhibition of acetylcholinesterase (AChE), the enzyme responsible for the breakdown of ACh in the brain, is the mode of action of the currently used, FDA approved anti-AD drugs, namely rivastigmine, galantamine and donepezil.

Isatin (2,3-dioxoindole) is considered a privileged scaffold for the design and synthesis of multifunctional molecules against neurodegenerative diseases. It has demonstrated potential inhibitory activity against neurodegeneration-related enzymes (cholinesterase, carbonic anhydrase, MAO-B), protein aggregation disturbance, neuroprotective properties and antioxidant activity [15,16,17,18]. Consequently, a variety of structural modifications have been investigated to optimize the desired properties and maximize the number of modalities, with the majority of these aiming at dual anti-Aβ aggregation and anti-AChE combination [19,20,21,22,23,24].

**Figure 1:**
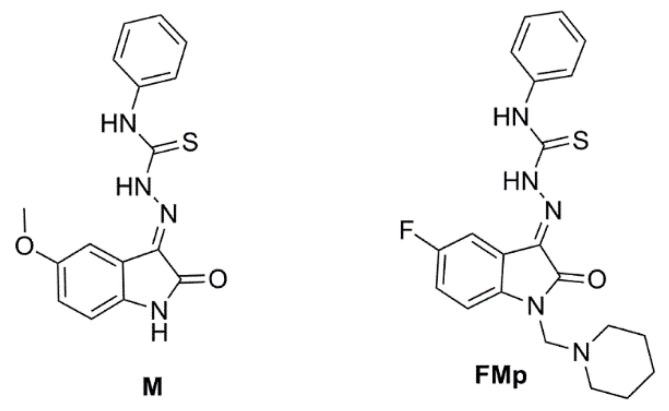
Chemical structure of the tested isatin thiosemicarbazones.

Based on all the aforementioned evidence, we would like to report herein on an investigation into the potentially prophylactic and multimodal mode of action of two isatin thiosemicarbazone (ITSC) derivatives, namely **M** and **FMp** (Figure 1). The compounds have been synthesized by our group and previously shown to inhibit Aβ aggregation [25]. In this work, the exogenous Aβ-mediated toxicity at different time points of pre- or post-incubation of the compounds in the SK-N-SH cell line were thoroughly investigated. The survival of both wild type and 5xFAD primary hippocampal cell cultures was also investigated upon treatment with the ITSCs. Furthermore, the levels of phosphorylation of GSK-3 and AKT in both wild type and 5xFAD-derived hippocampal cells were measured. Finally, the in vitro inhibition of acetyl cholinesterase, lipoxygenase and lipid peroxidation were assessed to evaluate the anti-cholinesterase, antioxidant and anti-inflammatory potential of the compounds.

## 2. Materials and Methods

### 2.1. Synthesis and Characterization

The ITSCs derivatives M and FMp were synthesized in high yielding reactions and fully characterized by spectroscopic methods (IR, MS and NMR), as previously described [25].

### 2.2. Cell Viability Assay (MTT)

Cell viability was determined in primary cultures after treatment with ITSCs (1 μΜ) for 24 h and in human neuroblastoma SK-N-SH cells after treatment with ITSCs/Aβ42 solutions at various experimental conditions as stated in the figure legends. The cells were incubated with MTT solution (1 mg/mL) for 4 h at 37 °C. Subsequently, formazan crystalline precipitates were resolubilized in DMSO and absorption was measured at 570 nm in a microtiter plate reader. The SK-N-SH cell line was obtained from the cell bank of the Institute of Biosciences & Applications, NCSR “Demokritos”.

### 2.3. Cell Culture, Cell Lysis and Immunoblotting

SK-N-SH cells were cultured in RPMI medium supplemented with 10% FBS at 37 °C and 5% CO_2_ before treatment with ITSCs and Aβ42, as stated in the figure legends. Primary hippocampal cells were isolated post-mortem from p0 newborn mice, cultured in Neurobasal A medium containing 2% B27 supplement at 37 °C, in a 5% CO_2_ atmosphere and were left to mature for 8 days prior to treatment with M and FMp (1 μM) for 24 h. Following treatment, primary cells were lysed in RIPA buffer (Tris-HCl 50 mM, NaCl 150 mM, EDTA 1 mM, Na deoxycolate 0.25%, TritonX-100 1%, SDS 0.1%) containing a protease and phosphatase inhibitors cocktail at 4 °C. Cell Lysates were separated by SDS-PAGE electrophoresis and then immunoblotted with antibodies against the phosphorylated forms of AKT, GSK-3β, and the housekeeping genes β-Tubulin or β-Actin. To identify 5xFAD mice genotyping was performed by PCR analysis of tail DNA using primers specific for human APP gene [26].

### 2.4. Preparation of Aβ Stock and Solutions

Aβ42 was gently dissolved without vortexing in Type 1 (Milli-Q) water to prepare a 10 μM stock solution for cell viability measurements. Solutions of Aβ42 in phosphate buffer (PB, 10 mM, pH 7.33) were prepared by adding the appropriate volumes of PB to aliquots of the aqueous stock solution to achieve the desired final concentration. To obtain oligomeric cytotoxic forms of Aβ42, solutions of the peptide alone, or in a mixture with ITSCs (stock concentration of 10 mM in DMSO), were allowed to stay at 37 °C for 24 h before use. The final DMSO concentration in the cell cultures was equal to 0.1% *v*/*v*.

### 2.5. Soybean LOX Inhibition Study In Vitro

The tested compounds dissolved in DMSO were incubated at room temperature with sodium linoleate (0.1 mL) and 0.2 mL of enzyme solution (1/9 × 10^−4^ *w*/*v* in saline) in 0.01 M Tris buffer pH 9. The conversion of sodium linoleate to 13-hydroperoxylinoleic acid at 234 nm was recorded and compared with the appropriate standard inhibitor NDGA [27].

### 2.6. Inhibition of Linoleic Acid Lipid Peroxidation

Sodium linoleate solution (10 μL, 16 mM) was added to the UV cuvette containing 930 μL of 0.05 M phosphate buffer, pH 7.4 pre-thermostated at 37 °C, under air, followed by the addition of AAPH solution (50 μL, 40 mM) which was used as a free radical initiator. Aliquots of the appropriate concentration of the tested compounds (10 μL) were added to the mixture, whereas DMSO was used as a blank for the measurement of lipid oxidation. The rate of oxidation at 37 °C was monitored by recording the increase in absorption at 234 nm caused by conjugated diene hydro peroxides. The results were compared to the appropriate standard inhibitor Trolox (93%) [28].

### 2.7. Inhibition of Acetyl-Cholinesterase

Thiol ester acetylthicholine (0.01 M) was used as a substrate for AChE (3.5 U/mL) which was hydrolyzed to produce thiocholine and acetate. The thiocholine reduced DTNB (0.01 M in phosphate buffer 0.1 M pH 8) liberating nitrobenzoate, which absorbed at 405 nm. The absorbance was measured with Perkin-Elmer multilabel plate reader Victor X3. The effect of ITSCs was assessed by recording the 405 nm absorption after the addition of an appropriately diluted stock solution (10 mM in DMSO) to the desired final concentration (100 µM) in phosphate buffer pH 8 (0.1 M). Tacrine was used as the reference compound [28].

### 2.8. Statistical Analysis

Data in all assays were the mean of at least three independent experiments. Graphs were analyzed using GraphPad Prism 5.0 software. In cell culture experiments the statistical significance of changes in different groups was evaluated by one-way analysis of variance (ANOVA) and Student’s t-tests. For each experiment, data were expressed as the mean ± standard error (sd) of the mean (SEM), * *p* ≤ 0.05, ** *p* ≤ 0.01, *** *p* ≤ 0.001, ns (not significant) > 0.05 compared to Aβ42 (1 μM) treatment and # *p* < 0.01 and ## *p* < 0.01, ### *p* < 0.001 compared to control (untreated cells).

## 3. Results and Discussion

### 3.1. ITCSs Rescue SK-N-SH Neuroblastoma Cells from Aβ-Induced Toxicity in a Prophylactic Manner

**Figure 2:**
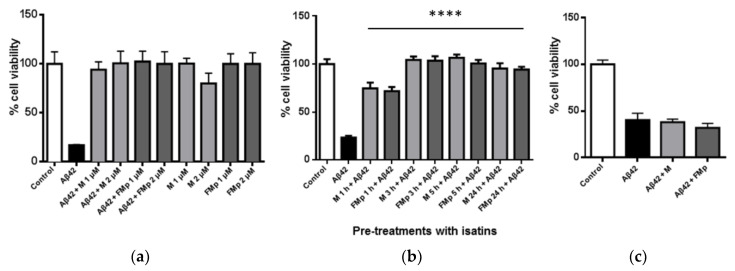
ITSC derivatives effectively protect against neurotoxicity caused by Aβ42 in SK-N-SH cells: (**a**) Mixtures of either M or FMp (1 and 2 μΜ) with Aβ42 (1 μΜ) were incubated for 24 h at 37 °C and then added to cells for 24 h. Negative control cells were treated with ITSCs alone in the same conditions. (**b**) Cells were treated with either M or FMp (1 μM) for 1, 3, 5 and 24 h prior to the addition of Aβ42 (1 μΜ) for 24 h. (**c**) Cells were treated for 24 h with 1 μΜ of Aβ42 followed by a 24 h treatment with 1 μΜ of M or FMp. Significance relative to control (**a**) and to Aβ42 (**b**,**c**). Results were expressed as the mean ± s.d. for at least three independent experiments and analyzed using one-way ANOVA (**** *p* < 0.0001).

Having shown that they can effectively inhibit Aβ aggregation, and aspired to explore in more detail the cell rescuing potential of **M** and **FMp** against Aβ-induced toxicity, three different cases were evaluated. Firstly, mixtures of **M** or **FMp** (1 or 2 μΜ) with Aβ42 (1 μΜ), pre-incubated at 37 °C for 24 h, were added to the SK-N-SH cells. When cells were treated with the 24 h-matured Aβ42 alone (1 μΜ) only a 20% cell survival was observed (Figure 2a). When ITSCs were used the cell viability was found to be nearly equal to control levels (non-exposed to Ab cells). This was a remarkable recovery of cell survival caused by ITCSs. These results suggested that ITSCs could completely rescue cells from Aβ toxicity, regardless of the severity of Aβ-induced toxicity and the cell type. As the compounds were pre-incubated with the Aβ42 peptide the apparent cell rescuing should be the result of the inhibition of Aβ aggregation by **M** and **FMp** [25]. This type of cell viability recovery has been previously reported for other isatin-related derivatives and other families of synthetic or natural molecules capable of inhibiting Aβ aggregation, or altering its pathway and, thus, reducing the toxic oligomeric entities [19,20,21,22,23,24,29,30,31,32,33].

Interestingly, very similar results were obtained when SK-N-SH cells were treated with either **M** or **FMp** for 1, 3 and 5 h prior to the addition of Aβ42 species. Figure 2b shows that although Aβ42 resulted in the same extent of cell cytotoxicity, ITCSs exhibited a time-dependent cell rescuing effect. More specifically, even after only one hour of cell pre-incubation with either **M** or **FMp,** nearly 50% recovery in cell survival was achieved. Moreover, both ITSCs resulted in complete cell rescuing upon pre-incubation of cells for more than 3 h prior to Aβ42 addition. To the best of our knowledge, such a prophylactic effect has not been reported for other isatin derivatives. This finding may be related to the previously reported antioxidant potential of the two compounds [25] potentially leading to augmentation of cellular antioxidant defense capacity. It can, therefore, be suggested that ITSCs not only act as inhibitors of the Aβ aggregation process but also contribute in the regulation of other pathways able to promote cell survival and/or to counterbalance the unwanted Aβ-induced effects.

Finally, when cells were treated with either of the ITSC derivatives after a 24 h-treatment with Aβ42, cell viability was not recovered and no cell protection was observed (Figure 2c). Therefore, post-Aβ cellular damage and cell death cannot be reversed by this type of ITSC agent.

### 3.2. ITSCs Restore Aβ-Induced Reduction in Ser473 Phosphorylation of Akt and in Ser9 Phosphorylation of GSK-3b in Primary Hippocampal Cells

**Figure 3:**
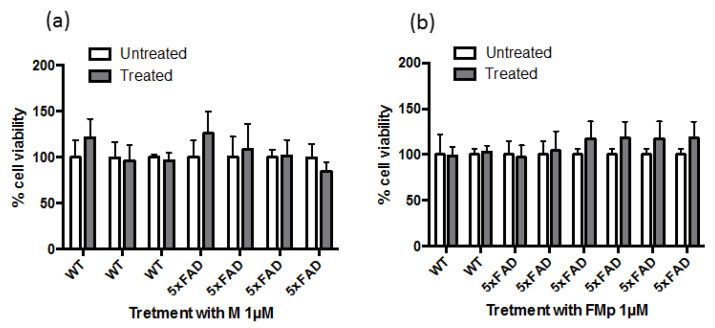
Cell viability of primary hippocampal cells upon treatment with ITSCs. Cells isolated from wild type (WT) and 5xFAD mice were treated for 24 h with 10 μM of **M** (**a**) and **FMp** (**b**). Results were expressed as the mean ± s.d. for at least three independent experiments and analyzed using two-way ANOVA.

Inhibition of the PI3K/Akt/GSK-3β signaling pathway is believed to be an important mechanism underlying the action of many neuroprotective agents [34]. Intrigued by the prophylactic effect observed in SK-N-SH cells, when the cells were pre-incubated with the agents and then treated with the Aβ species, the rescuing effect of the ITSC derivatives in this pathway was evaluated using primary hippocampal cultures derived from AD mice (5xFAD), which exerted more physiologically-relevant Aβ levels. Firstly, the cell viability upon treatment with **M** and **FMp** was measured by the MTT assay. As demonstrated in Figure 3 no statistically significant differences in cell viability between untreated and treated cells of either genotype were observed.

**Figure 4:**
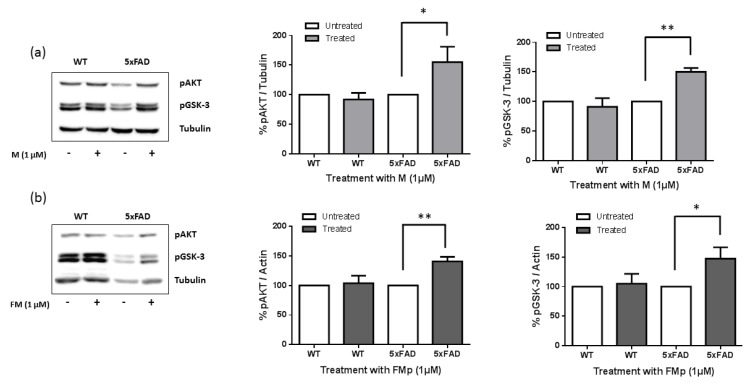
ITSCs derivatives reverse the inhibition of Akt and GSK-3β phosphorylation in 5xFAD cells. Primary hippocampal cells isolated from wild type (WT) and 5xFAD mice were treated for 24 h with (1 μM) of **M** (**a**) and **FMp** (**b**). Equal amounts of total protein were analyzed on 10% SDS-PAGE gels and immunoblotted with primary antibodies against phosphorylated AKT and GSK-3β kinases. Graphs depict densitometric quantification of phosphorylated proteins normalized to their housekeeping gene β-Tubulin or β-Actin. Results are expressed as the mean ± s.d. for at least three independent experiments and analyzed using a student t test (* *p* < 0.05, ** *p* < 0.01).

Cells were subsequently exposed to ITSCs (1 μΜ) and the levels of phosphorylated Akt at Ser473 and its downstream target GSK-3β at Ser9 were measured. The Western blotting results in Figure 4 (see also Figure S1 in Supporting Information) clearly demonstrate that initially 5xFAD cells were characterized by reduced levels of phosphorylation of both Akt and GSK-3β. This is in agreement with previous reports which connect the Aβ oligomers with the inhibition of the pro-survival (PI3K)/Akt signaling pathway and the overactivation of GSK-3β inducing neuronal death in vitro [26,35] Surprisingly, both compounds, even at the low micromolar dose, managed to reverse the inhibition of Akt phosphorylation at Ser473 and of GSK-3β phosphorylation at Ser9 sites observed in the 5xFAD cells. causing a nearly 50% increase in the phosphorylated protein load (Figure 4). This result may provide an explanation, at least partially, to the prophylactic and cell rescuing effect observed upon pre-treatment of the SK-N-SH cells with ITSCs. The pre-treatment of cells with ITSCs may cause the necessary deactivation of GSK-3β, along with possibly modulating other pro-survival signals, in a manner that cannot be reversed by the toxic and proapoptotic activity of Aβ.

Finally, since Alzheimer’s disease is considered to be a complex multifactorial disorder, the vast amount of research focuses on the development of multimodal agents towards all the currently known pathways. Our compounds were evaluated for their anti-cholinesterase activity. They exhibited a moderate ability to inhibit the Acetylcholinesterase enzyme with an IC_50_ value of approximately 100 μM. However, their antioxidant capacity was of higher potential, as witnessed by the effective inhibition of lipid peroxidation at 100 μM, in addition to their previously reported cellular ROS scavenging potential [25]. Moreover, a moderate anti-inflammatory activity was observed by recording an IC_50_ value of 60.5 and 54 μM, respectively, for **M** and **FMp**, against lipoxygenase activity (Table 1).

Finally, both compounds fulfilled the Lipinski’s rule of five for drugability, absorption and penetration, which states that molecules poorly absorbed by the intestinal wall present two or more of these characteristics: molecular weight > 500, the calculated logarithm of n-octanol/water partition coefficient (ClogP) > 5, more than 5 hydrogen-bond (HB) donor groups (expressed as the sum of OHs and NHs groups) and more than 10 HB acceptor groups (expressed as the sum of Os and Ns atoms) [36]. Indeed, the tested compounds have a MW < 500, number of hydrogen donor atoms < 5, number of acceptors < 10. In addition, the theoretically calculated Molinspiration lipophilicity values (miLogP) for compound **M** was at the range of 2.58 and for **FMp** the corresponding value was equal to 2.71. Furthermore, the Topological Polar Surface Area (TPSA) for **M** was 78.51 Å^2^ and for **FMp** it was 61.66 Å^2^ [37]. Bearing in mind that, for sufficient Blood brain Barrier penetration, the desirable lipophilicity (LogP) values are in the range of 1.5–2.7 and the upper TPSA limit is around 90 Å^2^ it appears that both molecules present favorable pharmacokinetic and BBB penetration properties and have the potential to exert their prophylactic and therapeutic effects at the site of Alzheimer disease pathology.

## 4. Conclusions

In conclusion, our results strongly suggest that the isatin thiosemicarbazone skeleton can be considered as a privileged structure with a potential multimodal activity against Alzheimer’s disease, beneficially affecting catalytic pathways and mechanisms to prevent neuronal death and degeneration.

## Figures and Tables

**Table 1:** In vitro inhibition of soybean lipoxygenase (IC_50_ μΜ), in vitro inhibition of acetyl-cholinesterase (IC_50_ μΜ or AChE Inh. %) and lipid peroxidation inhibition (LPI% at 100 μΜ).

Compound	LOX Inh. IC_50_ μΜ	% AChE inh @ 100 μΜ	LPI% @ 100 μΜ
**M**	60.5	54	77
**FMp**	54	44	51
**trolox**			92
**Tacrine**	-	98	
**NDGA**	0.5	-	

Note: SD < 10%.

## Supplementary Materials

The following supporting information can be downloaded at: https://www.mdpi.com/article/10.3390/brainsci12060806/s1, Figure S1: Full-length Western blot images.
