# Supplementary material for: The Prophylactic and Multimodal Activity of Two Isatin Thiosemicarbazones against Alzheimer’s Disease In Vitro
Source: Brain Sci. 2022 Jun 19;12(6):806. doi: 10.3390/brainsci12060806 (PMC9221192; doi:10.3390/brainsci12060806)
Supplement: Supplementary file 1 [file brainsci-12-00806-s001.zip › brainsci-1742534 - SI.pdf]

The prophylactic and multimodal activity of two isatin thiosemicarbazones against Alzheimer's disease in vitro

Barbara Mavroidi[1], Archontia Kaminari[1*], Dimitris Matiadis[1], Dimitra Hadjipavlou-Litina[2], Maria Pelecanou[1], Athina Tzinia[1], Marina Sagnou[1*]

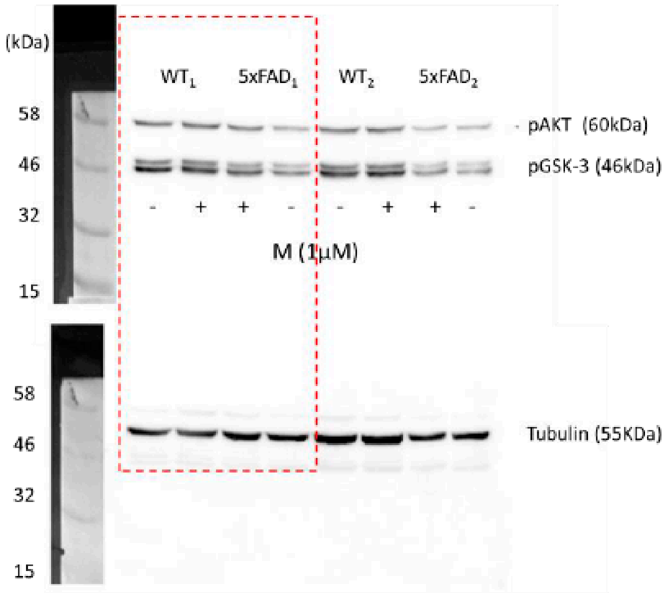

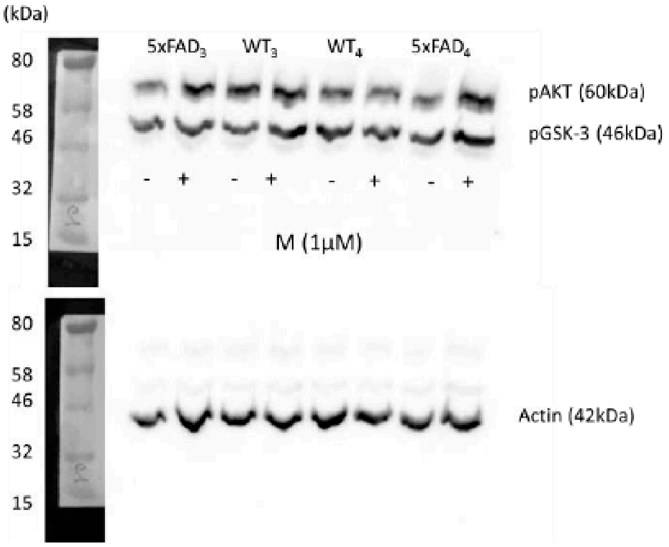

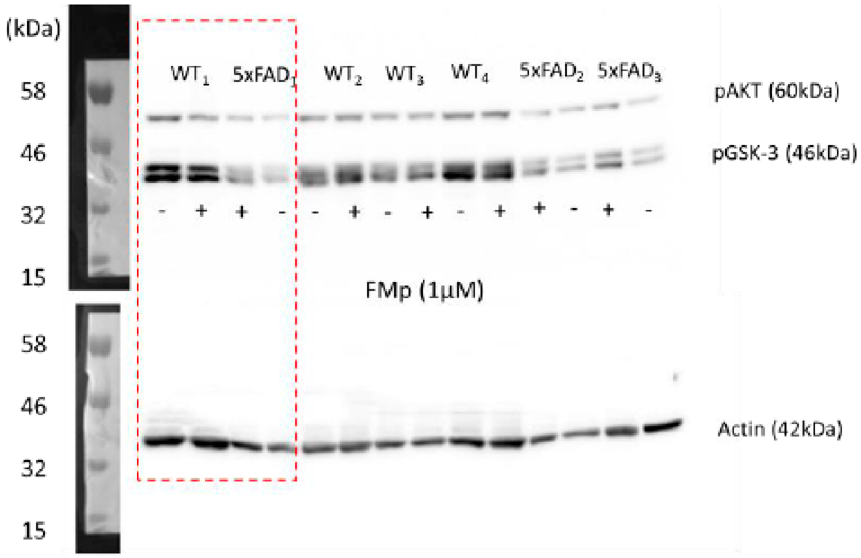

Figure S1: Full-length western blots. Red brackets depict samples selected as representative results from 3 to 4 biological replicates per Isatin treatment. Please note that selected 5xFAD samples have been inverted in the final text for presentation purposes.
